# Exploring the lncRNAs Related to Skeletal Muscle Fiber Types and Meat Quality Traits in Pigs

**DOI:** 10.3390/genes11080883

**Published:** 2020-08-04

**Authors:** Rongyang Li, Bojiang Li, Aiwen Jiang, Yan Cao, Liming Hou, Zengkai Zhang, Xiying Zhang, Honglin Liu, Kee-Hong Kim, Wangjun Wu

**Affiliations:** 1 Department of Animal Genetics, Breeding and Reproduction, College of Animal Science and Technology, Nanjing Agricultural University, Nanjing 210095, China; 2018205007@njau.edu.cn (R.L.); 2019205006@njau.edu.cn (A.J.); 2018205001@njau.edu.cn (Y.C.); liminghou@njau.edu.cn (L.H.); 2017105031@njau.edu.cn (Z.Z.); 2018105030@njau.edu.cn (X.Z.); wangjunwu4062@163.com (H.L.); 2 College of Animal Science and Veterinary Medicine, Shenyang Agricultural University, Shenyang 110866, China; libojiang12@126.com; 3 Department of Food Science, Purdue University, West Lafayette, IN 47897, USA; keehong@purdue.edu

**Keywords:** pig, skeletal muscle fiber, meat quality, metabolic diseases, lncRNA, RNA-seq

## Abstract

The alteration in skeletal muscle fiber is a critical factor affecting livestock meat quality traits and human metabolic diseases. Long non-coding RNAs (lncRNAs) are a diverse class of non-coding RNAs with a length of more than 200 nucleotides. However, the mechanisms underlying the regulation of lncRNAs in skeletal muscle fibers remain elusive. To understand the genetic basis of lncRNA-regulated skeletal muscle fiber development, we performed a transcriptome analysis to identify the key lncRNAs affecting skeletal muscle fiber and meat quality traits on a pig model. We generated the lncRNA expression profiles of fast-twitch *Biceps femoris* (Bf) and slow-twitch *Soleus* (Sol) muscles and identified the differentially expressed (DE) lncRNAs using RNA-seq and performed bioinformatics analyses. This allowed us to identify 4581 lncRNA genes among six RNA libraries and 92 DE lncRNAs between Bf and Sol which are the key candidates for the conversion of skeletal muscle fiber types. Moreover, we detected the expression patterns of lncRNA *MSTRG.42019* in different tissues and skeletal muscles of various development stages. In addition, we performed a correlation analyses between the expression of DE lncRNA *MSTRG.42019* and meat quality traits. Notably, we found that DE lncRNA *MSTRG.42019* was highly expressed in skeletal muscle and its expression was significantly higher in Sol than in Bf, with a positive correlation with the expression of *Myosin heavy chain 7* (*MYH7*) (*r* = 0.6597, *p* = 0.0016) and a negative correlation with meat quality traits glycolytic potential (*r* = −0.5447, *p* = 0.0130), as well as drip loss (*r* = −0.5085, *p* = 0.0221). Moreover, we constructed the lncRNA *MSTRG.42019*–mRNAs regulatory network for a better understanding of a possible mechanism regulating skeletal muscle fiber formation. Our data provide the groundwork for studying the lncRNA regulatory mechanisms of skeletal muscle fiber conversion, and given the importance of skeletal muscle fiber types in muscle-related diseases, our data may provide insight into the treatment of muscular diseases in humans.

## 1. Introduction 

Skeletal muscle is the major component of body mass accounting for approximately 50% of body mass in a mammal, and its growth and development are critical for maintaining skeletal muscle function. Skeletal muscle is composed of various muscle fibers that exhibit different physiological and metabolic properties, such as glycolysis, oxidative metabolism, and contraction [1]. Notably, dysfunctions of skeletal muscle fiber types are known to many human diseases, such as muscular dystrophy, cardiomyopathic disease, and type 2 diabetes [2,3,4,5]. Moreover, the differences in skeletal muscle fiber types directly affect meat quality postmortem in livestock, such as pH, meat color, and drip loss [6]. Therefore, elucidating the underlying mechanisms of skeletal muscle growth and development and skeletal muscle fibers formation will be useful for the treatment of human muscle diseases and the improvement of production traits of livestock.

Myogenesis is a complex process that is controlled by a series of factors, such as myogenic regulatory factors (MRFs) [7,8] and non-coding small molecule RNA microRNAs (miRNAs) [9,10]. In recent years, emerging researches found a key role of long non-coding RNAs (lncRNAs) in skeletal muscle growth and development and found that they are closely related to muscle diseases [11,12]. LncRNAs are a class of non-coding RNAs with a length of more than 200 nt and mainly transcribed by RNA polymerase II in eukaryotic organisms [13]. For example, Cesana et al. found that lncRNA linc-MD1 is expressed explicitly in the differentiating myoblasts and exerts its function through miR-133 and miR-135-regulated expression of muscle-specific transcriptional regulators, MAML1 and MEF2C [14]. Dey et al. found that lncRNA lncRNA-H19 controls skeletal muscle differentiation and regeneration by two miRNAs generated from its own exon 1 [15]. Moreover, it has been reported that lncRNAs affect skeletal muscle growth and development by regulating DNA methylation [16]. Intriguingly, researchers recently found that a skeletal muscle-specific RNA, annotated as a putative the lncRNA RNA, exerts its role in skeletal muscle physiology via encoding a conserved micropeptide [17].

Notably, the types of skeletal muscle fiber are critical factors related to various metabolic diseases in humans and affecting meat quality traits in livestock, but the researches on the genetic basis of skeletal muscle fiber formation involved in lncRNAs are still limited. In this study, we performed a comparative analysis of the whole transcriptomes between *Biceps femoris* (Bf) (fast muscle or white muscle) and *Soleus* (Sol) (slow muscle or red muscle) muscles characterized by noticeable muscle fiber type differences using RNA-seq. We further identified a series of differentially expressed (DE) lncRNAs between Bf and Sol muscles, which represent potential candidate lncRNAs affecting the conversion of skeletal muscle fiber types. Furthermore, we found several important Gene Ontology (GO) terms and metabolic pathways associated with skeletal muscle fiber formation. We proposed that lncRNA *MSTRG.42019* may be a key lncRNAs affecting skeletal muscle fiber types based on its expression profiles and relationship with porcine meat quality traits and its network interaction map. Overall, our data provide the groundwork for an understanding of the lncRNA-regulated skeletal muscle growth and development and muscle fiber conversion.

## 2. Materials and Methods

### 2.1. Ethics Statement

All experimental procedures on the pigs were conducted according to the Guidelines of the Institutional Animal Care and Use Committee of Nanjing Agricultural University, Nanjing, China (SYXK2011-0036).

### 2.2. Animals and Samples Collection

Three full-sibling Duroc × Meishan female pigs derived from the offspring of a Duroc boar crossing with eight Meishan sows were used in this study and the experimental pig population was constructed by Li et al. [18]. Two types of skeletal muscles with different meat color, Bf (Bf28, Bf35, and Bf36) and Sol (Sol28, Sol35, and Sol36) muscles, were dissociated from these three full-sibling Duroc × Meishan pigs. Previously, we have only identified the DE coding genes due to the limitation of the library construction method and sequencing depth [18]. In this study, we reconstructed the RNA sequencing library to identify the DE coding and non-coding genes using the Bf (Bf28, Bf35, and Bf36) and the Sol (Sol28, Sol35, and Sol36) muscles. Moreover, twenty *Longissimus dorsi* muscles from a 279 commercial hybrid pig population [Pietrain (P) × Duroc (D)] × [Landrace (L) × Yorkshire (Y)] were selected randomly to detect the correlation between the expression of DE lncRNAs and meat quality traits, and the experimental pigs and the production traits were described previously by Dong et al. [19].

### 2.3. RNA Isolation and LncRNA Library Construction

Total RNA was extracted using the Trizol^®^ reagent (Invitrogen, Carlsbad, CA, USA), and the quality of the isolated RNA was evaluated by 1% agarose gels. The purity of total RNA was initially detected using the Kaiao K5500^®^ Spectrophotometer (Kaiao, Beijing, China). Next, RNA integrity and concentration were precisely assessed using the RNA Nano 6000 Assay Kit of the Agilent Bioanalyzer 2100 (Agilent Technologies, Santa Clara, CA, USA). LncRNA libraries were constructed using 3 μg total RNA per sample. Ribo-Zero^TM^ Gold Kits (Ribo, Guangzhou, China) was used to remove the ribosomal RNA (rRNA) from the sample, and the different indexes were used to build the libraries according to the NEBNext^®^ Ultra^TM^ Directional RNA Library Prep Kit for Illumina^®^ (NEB, Ipswich, MA, USA). The specific steps for the library construction were as follows: firstly, rRNA was removed from total RNA and the first-strand cDNA was synthesized with fragmented RNA using a six-base random primer. Next, the second-strand cDNA was synthesized by adding buffer, dNTPs, RNase H, and DNA Polymerase I. Subsequently, the library fragments were recovered by agarose gel electrophoresis after purification using the QIAQuick PCR kit (Qiagen, New York, NY, USA), followed by the end-repair, adding base A and adapter ligation. Finally, the double cDNA strand was digested with uracil-N-glycosylase (UNG) enzyme and subjected to PCR amplification, and the 300 bp target fragments were recovered by gel electrophoresis to obtain the sequencing libraries. The RNA concentration of libraries was measured using the Qubit^®^ RNA Assay Kit (Thermo Fisher, New York, NY, USA) in Qubit^®^ 2.0 (Thermo Fisher, New York, NY, USA), and the size of the inserted fragment was assessed using Agilent Bioanalyzer 2100 (Agilent Technologies, Santa Clara, CA, USA). After cluster generation, sequencing was performed on an Illumina Hiseq 4000 sequencing platform with 150 bp paired-end reads. The raw data of the transcriptome sequence data have been deposited in NCBI SRA (accession codes PRJNA597666).

### 2.4. Quality Control and Genomic Alignment

Raw data were processed with Perl scripts to obtain high-quality clean data for further analyses. The filtering criteria were as follows: (1) remove the adaptor-polluted reads (reads containing more than 5 adapter-polluted bases were regarded as adaptor-polluted reads and were filtered out); (2) remove the low-quality reads (reads with a Phred quality value of less than 19 accounting for more than 15% of total bases are regarded as low-quality reads); (3) remove reads whose number of N bases account for more than 5%. As for paired-end sequencing data, both reads were filtered out if any reads of the paired-end reads were adaptor-polluted. The pig reference genome (*Sscrofa11.1*) and the annotation file were downloaded from the Ensembl database (http://www.ensembl.org/index.html). Bowtie2 v2.2.3 was used for building the genome index [20], and clean data were mapped to the reference genome using HISAT2 v2.0.5 [21].

### 2.5. Identification of LncRNAs

Novel transcripts were reconstructed using the StringTie software (v1.3.3b) with the default parameters using the mapped clean reads [22], and GffCompare was used to screen out known mRNAs and other non-coding RNAs (rRNA, tRNA, snoRNA, snRNA, known lncRNA, etc.). Furthermore, the known lncRNAs were also figured out through comparative analysis. Subsequently, the novel potential lncRNAs transcripts were identified according to the transcript length (≥200 bp), the exon number (≥2), and the read coverage of each transcript (≥5). Then, we used four kinds of tools to predict the protein-coding potential of novel transcripts to confirm novel lncRNAs, including the Coding-Non-Coding Index (CNCI) [23], Coding Potential Calculator (CPC) [24], PFAM protein domain analysis [25,26], and the Coding Potential Assessment Tool (CPAT) [27]. Finally, the co-existing transcripts were designated as novel lncRNAs. Moreover, we performed structure and expression characteristic analyses of the novel lncRNAs and known lncRNAs.

### 2.6. Differentially Expressed Gene Analyses

HTseq v0.6.0 [28] was utilized to calculate the read count for each gene, and RPKM (Reads Per Kilobase Millon Mapped Reads) was used to estimate the expression level of genes in each sample. The formula is shown as

(1) $$\mathrm{RPKM}\;=\frac{10^{6}\times R}{\;\mathrm{NL}/10^{3}}.$$

*R* represents the number of reads assigned to a specific gene in each sample, *N* represents the total number of mapped reads in each sample, and L represents the length of a specific gene. Furthermore, DEseq2 [29] was used to identify the differentially expressed genes (DEGs), including protein-coding and non-coding genes, between Bf and Sol groups. DEGSeq (v1.16) [30] was used to determine the DEGs between two paired samples (Bf28 vs. Sol28; Bf35 vs. Sol35; Bf36 vs. Sol36). The *p*-value was adjusted by the Benjamini and Hochberg’s approach to control the false discovery rate, and *q* ≤ 0.05 and |log2_ratio| ≥ 1 were identified as DEGs.

Furthermore, nine lncRNAs were selected for validating the RNA-seq results using qRT-PCR. cDNA was synthesized with the total RNA derived from the same muscle samples for RNA-seq with the Takara PrimeScript^TM^ II 1st Strand cDNA Synthesis Kit (TaKaRa, Dalian, China), and qRT-PCR was performed on a Step-One Plus Real-Time PCR System (Applied Biosystems, Carlsbad, CA, USA) using the AceQ qPCR SYBR Green Master Mix (Vazyme, Nanjing, China). The primers for quantitative analysis were shown in Additional File 5: Table S1. Relative expression levels were calculated using the 2^−ΔΔct^ value method [31] and porcine *GAPDH* was used for normalization of lncRNAs expression as an endogenous reference gene.

### 2.7. Target Prediction of DE LncRNAs and GO and KEGG Enrichment Analyses

The Spearman correlation coefficient between the expression of mRNAs and DE lncRNAs was calculated and the mRNAs with high a Spearman correlation coefficient (r ≥ 0.90 or ≤ −0.90) were selected as the trans-targets of DE lncRNAs, while the mRNAs with a distance of less than 50 kb to DE lncRNAs were selected as the cis-targets based on the possible region of target genes of lncRNAs [32,33]. To understand the biological functions of the target genes of DE lncRNAs, we performed the GO (Gene Ontology, http://geneontology.org/) and KEGG (Kyoto Encyclopedia of Genes and Genomes, http://www.kegg.jp/) enrichment analyses. The GO and KEGG enrichment of target genes of DE lncRNAs was implemented by the hypergeometric test in which the *p*-value was adjusted by multiple comparisons as *q*-value. The GO and KEGG terms with *p* < 0.05 were considered to be significantly enriched.

### 2.8. Expression Patterns of LncRNA MSTRG.42019

Among these DE lncRNAs, we found that lncRNA *MSTRG.42019* was a promising DE lncRNA affecting skeletal muscle growth and development and muscle fiber types. To validate its expression pattern, we detected its expression levels in Bf and Sol and analyzed its expression patterns in different tissues of adult pigs and 70-day-old fetuses (P70) and *Longissimus dorsi* muscles derived from different developmental stages, including 110-day-old fetuses (P110), the day of birth (D0), and 70 days after birth (D70). Relative expression levels of lncRNA *MSTRG.42019* were calculated using the 2^−ΔΔct^ value method [31] and normalized using porcine *GAPDH* as an endogenous reference gene.

### 2.9. Correlation Analyses of LncRNAs MSTRG.42019 and Meat Quality Traits

The skeletal muscle mass and muscle fiber types are closely related to porcine meat quality. To confirm the correlation between lncRNA *MSTRG.42019* and meat quality traits, we randomly selected 20 samples from 279 [(P) × (D)] × [(L) × (Y)] commercial pig populations and detected the expression levels of *Myosin heavy chain 7* (*MYH7*), *Myosin heavy chain 4* (*MYH4*), and *Myoglobin* (*MyoB*) using qRT-PCR and performed the correlation analyses between the expression of lncRNA *MSTRG.42019* and *MYH7*, *MYH4*, and *MyoB*. Furthermore, we performed the correlation analyses between the expression levels of lncRNA *MSTRG.42019* and meat quality traits, including carcass weight, back fat, intramuscular fat, pH_45min_, *L*_24h_, *a*_24h_, *b*_24h_, glycolytic potential, and drip loss.

### 2.10. Construction of LncRNA MSTRG.42019–mRNA Network

DEGs and the *trans*- or *cis*-targets of DE lncRNAs were derived from the RNA-seq data in this study, and the network, including DEGs and *trans*- or *cis*-targets of DE lncRNAs *MSTRG.42019*, was constructed using the Cytoscape software. The visualized network was edited based on the attribution of each gene [34]. Furthermore, we performed a pathway search for the DE target genes of lncRNA *MSTRG.42019* using the KEGG Mapper tool (https://www.kegg.jp/kegg/tool/map_pathway2.html).

### 2.11. Statistical Analyses

Statistical analyses were performed using the Prism 7 software (GraphPad Software, San Diego, CA, USA), and data were expressed as mean ± SE unless otherwise noted. Differences were tested using a two-tailed unpaired Student’s *t*-test or a one-way analysis of variance (ANOVA) with Duncan’s test. A *p*-value of <0.05 was considered significantly different.

## 3. Results

### 3.1. Generation of Transcriptome Data

In this study, we generated six RNA-seq libraries, namely, Bf28, Bf35, Bf36, Sol28, Sol35, and Sol36. The alignment results showed that the average ratio of rRNA mapping was less than 0.20%, and the ratio of clean Q30 bases (those with a base quality of >30 and an error rate of <0.001) in each library was higher than 94%, indicating high-quality raw reads from each library. After strict filtering, more than 15 Gb clean bases data corresponding to more than 100 million reads were obtained from each library after strick filtering, and the ratio of clean reads in each library reached more than 93%. The transcriptome sequence data generated from this study were deposited in NCBI SRA (accession code: PRJNA597666).

### 3.2. Novel LncRNAs Prediction and Characteristics Analyses

In this study, 5864 overlapped novel lncRNA transcripts without coding potential were identified using four kinds of predicted tools (Additional File 1: Figure S1). Detailed information for the coding potential of novel predicted lncRNAs are listed in Additional File 6: Table S2. Additional File 6: Table S2-1, Table S2-2, Table S2-3, and Table S2-4 represent the predicted results from CNCI, PFAM protein domain analysis, CPAT, and CPC, respectively. Moreover, our results showed that the exon number of lncRNAs was gathered at 2–4, while the exon number of mRNA was distributed in 1–29 (Additional File 2: Figure S2A). The length distribution showed that the length of lncRNAs was shorter than mRNA and mainly distributed in 200 bp and 2900 bp (Additional File 2: Figure S2B). In addition, our results indicated that the relative expression level of lncRNAs was lower than that of mRNA (Additional File 2: Figure S2C).

### 3.3. Identification of DE LncRNAs and Target Genes Prediction

**Figure 1:**
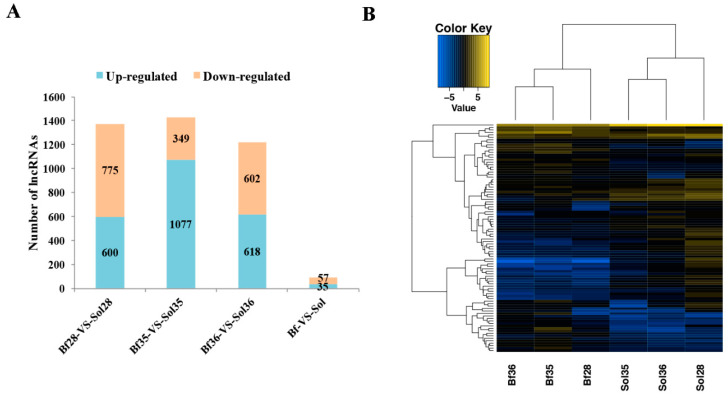
Statistics and heat map analyses of differentially expressed (DE) long non-coding RNAs (lncRNAs). (**A**) Statistics of DE lncRNAs. The X-axis represents the different compared groups, *Biceps femoris* (Bf) vs. *Soleus* (Sol) indicates the DE lncRNAs from the DEseq2 method; Bf28 vs. Sol28, Bf35 vs. Sol35, and Bf36 vs. Sol36 indicate DE lncRNAs from the DEGseq method. The Y-axis shows the number of DE lncRNAs. (**B**) Heat map analysis of DE lncRNAs between Bf and Sol muscles. Heat map analysis was conducted with 92 overlapped DE lncRNAs among three different comparative groups (Bf28 vs. Sol28, Bf35 vs. Sol35, and Bf36 vs. Sol36). Each column represents a sample and each row represents a DE lncRNA. Yellow and blue gradients indicate an increase and decrease in gene expression abundance, respectively.

Genomic expression analysis revealed that 4362 novel and 219 known lncRNA genes were detectable from Bf vs. Sol. In these, 3786 novel and 125 known lncRNA genes were detectable from Bf28 vs. Sol28, and 3741 novel and 126 known lncRNA genes were detectable from Bf35 and Sol35, and 3787 novel and 120 known lncRNA genes were detectable from Bf36 and Sol36 (Additional File 7: Table S3). Furthermore, 92 DE lncRNA genes were identified between Bf and Sol, including 35 up-regulated and 57 down-regulated lncRNAs (Figure 1A, Additional File 8: Table S4). Hierarchical clustering showed that lncRNA expression patterns between Bf and Sol were distinguishable (Figure 1B), indicating a substantial difference exists between the Bf and Sol groups, while a small variation exists among three biological replicates. Moreover, 600 up-regulated and 775 down-regulated lncRNA genes were identified in Bf28 vs. Sol28; 1077 up-regulated and 349 down-regulated lncRNA genes were identified in Bf35 vs. Sol35; and 618 up-regulated and 602 down-regulated lncRNA genes were identified in Bf36 vs. Sol36 (Additional File 8: Table S4). In addition, 246 DE coding genes were identified between Bf and Sol (Additional File 9: Table S5).

**Figure 2:**
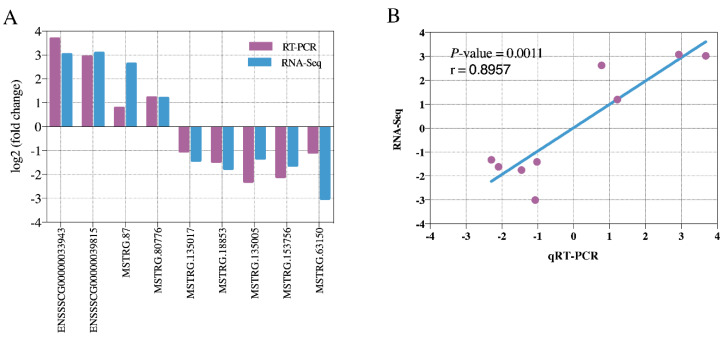
Validation of DE lncRNAs and correlation analysis. (**A**) Validation of DE lncRNAs by qRT-PCR (*n* = 3). Relative expression levels were calculated using the 2^−ΔΔct^ value method and porcine *GAPDH* was used for normalization of lncRNAs expression levels as an endogenous reference gene. The X-axis represents DE lncRNAs and the Y-axis represents the log_2_ (fold change) for qRT-PCR and RNA-Seq. (**B**) Correlation analysis of the expression of DE lncRNAs between qRT-PCR and RNA-Seq. The X and Y-axis represent the log_2_ (fold change) measured by qRT-PCR and RNA-Seq, respectively.

To validate the RNA-Seq results, nine DE lncRNAs, including two known lncRNAs and seven novel lncRNAs, were selected to confirm their expression differences between Bf and Sol by qRT-PCR assay. We found that the expression patterns of these lncRNAs were in agreement with that in RNA-Seq (Figure 2A) and the correlation coefficient of two methods was 0.8957 (*p* = 0.0011) (Figure 2B). These results indicate that DE lncRNAs identified from RNA-Seq were reliable. In addition, since lncRNAs exert their functions mainly via interaction with protein-coding genes through *cis* or *trans*, *cis* and *trans* target genes prediction of DE lncRNAs from Bf vs. Sol was performed. A total of 111 *cis* target genes and 9,050 *trans* target genes were predicted (Additional File 10: Table S6).

### 3.4. GO and KEGG Pathway Enrichment Analyses of Target Genes of DE LncRNAs

To understand the function of these DE lncRNAs, we performed the functional enrichment analyses for their target genes. GO analysis results showed that some interesting GO terms related to skeletal muscle fiber properties, such as lipid metabolic process, muscle system process, muscle contraction, muscle structure development, and ATP metabolic process, were significantly enriched in Biological Process (BP); and the mitochondrial part, mitochondrion, mitochondrial membrane part, respiratory chain complex, and mitochondrial membrane were significantly enriched in Cellular Component (CC); the catalytic activity, protein serine/threonine kinase activity, kinase activity, protein kinase activity, and calcium ion binding were significantly enriched in Molecular Function (MF) (Additional File 11: Table S7). KEGG pathway analysis showed that Alzheimer’s disease, Parkinson’s disease, Huntington’s disease, non-alcoholic fatty liver disease, and thermogenesis are the five most significantly enriched pathways; moreover, glycolysis/gluconeogenesis, cardiac muscle contraction, fatty acid metabolism, fatty acid degradation, and pyruvate metabolism pathways were also significantly enriched (Additional file 11: Table S7).

### 3.5. DE LncRNAs Affecting Muscle Fiber Types

**Figure 3:**
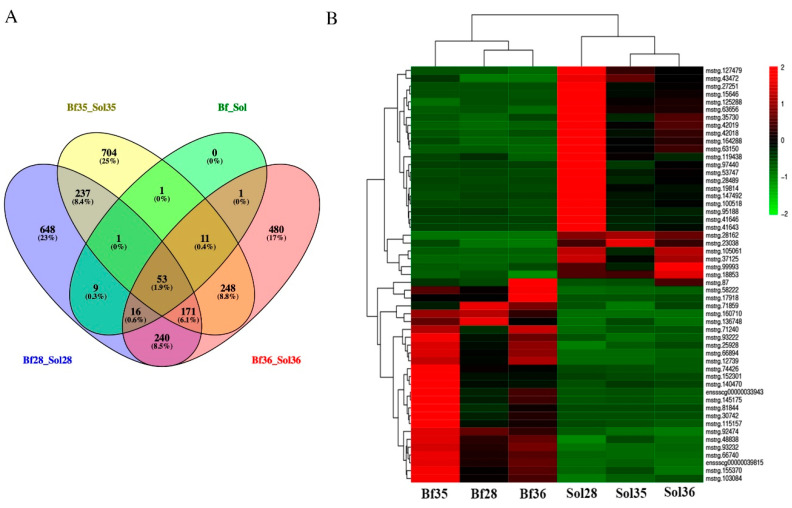
Overlapped and heat map analyses of DE lncRNAs. (**A**) Venn diagram of DE lncRNAs. A total of 53 overlapped DE lncRNAs were obtained from DEseq2 and DEGseq methods. Different color represents a different combination, and the number in the overlapped region represents the overlapped DE lncRNAs number. (**B**) Heat map analysis of 53 DE lncRNAs. Heat map analysis was conducted with 53 overlapped DE lncRNAs from four different comparative groups (Bf28 vs. Sol28, Bf35 vs. Sol35, Bf36 vs. Sol36, and Bf vs. Sol). Each column represents a sample and each row represents a DE lncRNA. Red and green gradients indicate an increase and decrease in gene expression abundance, respectively.

**Figure 4:**
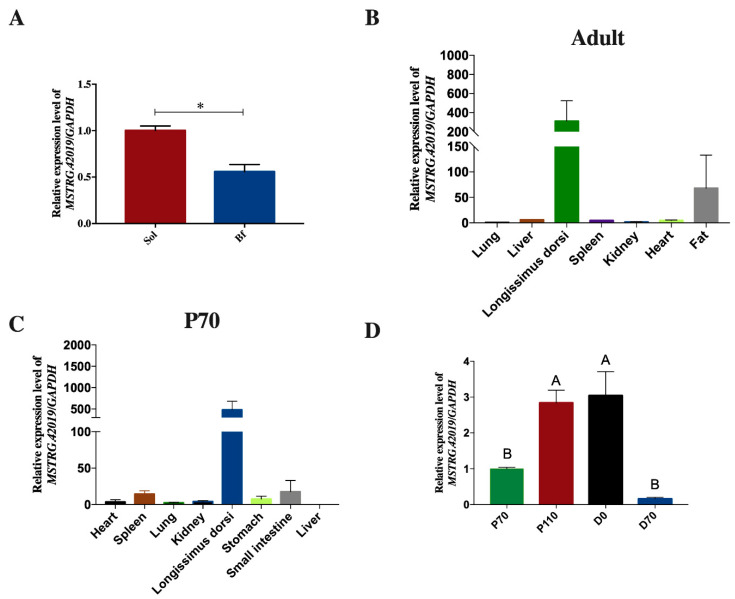
Expression patterns of lncRNA *MSTRG.42019*. (**A**) Expression of lncRNA *MSTRG.42019* in Bf and Sol muscles. (**B**) Expression profile of lncRNA *MSTRG.42019* in different tissues of adult pigs, *n* = 3. (**C**) Expression profile of lncRNA *MSTRG.42019* in different tissues of 70-day-old fetuses, *n* = 3. (**D**) Expression patterns of lncRNA *MSTRG.42019* in *Longissimus dorsi* muscles derived from different developmental stages, *n* = 3. Expression levels were determined by qRT-PCR and relative expression levels were calculated using the 2^−ΔΔct^ method and normalized to the expression of *GAPDH*. The expression of lncRNA *MSTRG.42019* in the sample of the first column was determined as control and normalized to 1. All data are presented as mean ± SE, and an unpaired Student’s *t*-test in the Prism 7 software was performed to evaluate significant differences between Bf and Sol. ANOVA with Duncan’s test was used to evaluate significant differences between groups P70 (70 days of pregnancy), P110 (110 days of pregnancy), D0 (the day of birth), and D70 (70 days after birth). * *p* ≤ 0.05, different letters above the bars indicate significant differences; *p* < 0.01.

In this study, 53 overlapped DE lncRNAs were filtered out from Bf28 vs. Sol28, Bf35 vs. Sol35, Bf36 vs. Sol36, and Bf and Sol (Figure 3A and Additional File 12: Table S8), and the heat map of 53 DE lncRNAs with distinguishable expression patterns is shown in Figure 3B. Among these DE lncRNAs, we noted that the lncRNA *MSTRG.42019* expression level was significantly higher in Sol muscle than that in Bf muscle (Figure 3B and Figure 4A) and was relatively highly expressed in skeletal muscle than that in other tissues in adult pig (Figure 4B and Figure S3A) and fetuses (Figure 4C). In addition, the expression patterns of lncRNA *MSTRG.42019* were shown to up-regulate before birth from 110 days of pregnancy (P110) to the day of birth (D0) and then down-regulated after birth (Figure 4D and Figure S3B).

### 3.6. Correlation between LncRNA MSTRG.42019 Expression and Meat Quality Traits

**Figure 5:**
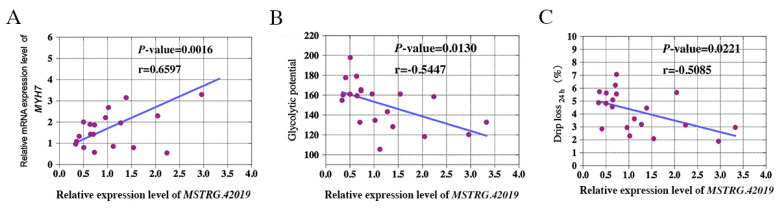
Correlation analyses between the expression of lncRNA *MSTRG.42019* and muscle fiber-related genes and meat quality traits. (**A**) Correlation between the expression of lncRNA *MSTRG.42019* and *Myosin heavy chain 7* (*MYH7*); (**B**) correlation between the expression of lncRNA *MSTRG.42019* and glycolytic potential of *Longissimus dorsi* muscles; (**C**) correlation between the expression of lncRNA *MSTRG.42019* and drip loss of *Longissimus dorsi* muscles. Twenty *Longissimus dorsi* muscles were derived from a 279 [(P) × (D)] × [(L) × (Y)] commercial hybrid pig population.

To elucidate the relationship between lncRNA *MSTRG.42019* and meat quality traits, we detected the expression levels of lncRNA *MSTRG.42019* and *MYH7*, *MYH4*, and *MyoB* in 20 *Longissimus dorsi* muscles derived from a 279 [(P) × (D)] × [(L) × (Y)] commercial hybrid pig population and performed correlation analyses between the expression of lncRNA *MSTRG.42019* and *MYH7*, *MYH4*, *MyoB*, and meat quality traits. Our results showed that expression levels of lncRNA *MSTRG.42019* were positively correlated with the mRNA expression level of *MYH7* (*r* = 0.659, *p* = 0.0016) (Figure 5A), with no correlation with the expression levels of *MyoB* and *MYH4* (Additional File 4: Figure S4A,B). Additionally, the expression level of lncRNA *MSTRG.42019* was negatively correlated with glycolytic potential (*r* = −0.5447, *p* = 0.013) (Figure 5B) and drip loss (*r* = −0.5085, *p* = 0.0221) (Figure 5C), respectively. Furthermore, no correlation was observed between the expression of lncRNA *MSTRG.42019* and the carcass weight (Additional File 4: Figure S4C), back fat (Additional File 4: Figure S4D), pH_45min_ (Additional File 4: Figure S4E), *L*_24h_ (Additional File 4: Figure S4F), *a*_24h_ (Additional File 4: Figure S4G), *b*_24h_ (Additional File 4: Figure S4H), and intramuscular fat (Additional File 4: Figure S4I).

### 3.7. LncRNA MSTRG.42019–mRNA Interaction Network

**Figure 6:**
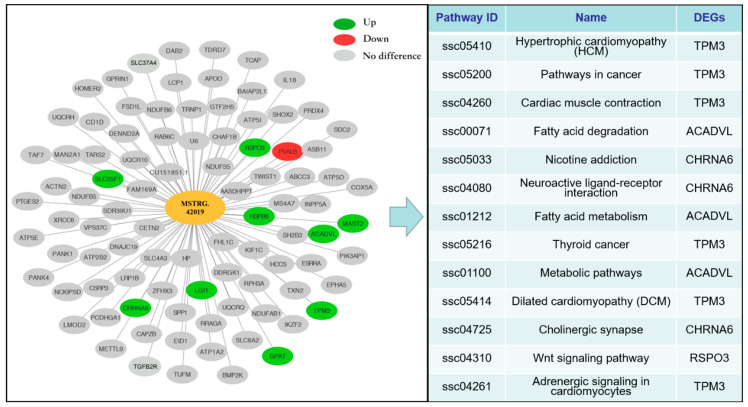
LncRNA *MSTRG.42019*–target mRNA interaction network and KEGG pathway search. Cytoscape was used to construct the interaction network between lncRNA *MSTRG.42019* and target mRNAs. Red and green represent down-regulated and up-regulated target genes from Bf and Sol, respectively, and gray indicates non-DE target genes. The KEGG pathway search for DE target genes was conducted using the website tool KEGG Mapper (https://www.kegg.jp/kegg/tool/map_pathway2.html).

To further explore the underlying function of lncRNA *MSTRG.42019*, we constructed a network map of lncRNA *MSTRG.42019* and its target genes and performed the KEGG pathway search for the DE target genes of lncRNA *MSTRG.42019* (Figure 6). The results showed that the *parvalbumin* (*PVALB*) was the down-regulated gene targeted by lncRNA *MSTRG.42019*. Moreover, the up-regulated expressed genes, including *Glycerol-3-phosphate acyltransferase* (*GPAT*), *Tropomyosin-3* (*TPM3*), *R-spondin 3* (*RSPO3*), *Heat shock protein family B member 6* (*HSPB6*), *Leucine-rich glioma inactivated 1* (*LGI1*), *Cholinergic receptor nicotinic alpha 6 subunit* (*CHRNA6*), *Solute carrier family 35 member F1* (*SLC35F1*), *Acyl-CoA dehydrogenase very long chain* (*ACADVL*), and *Microtubule*-*associated serine/threonine kinase 2* (*MAST2*) were also predicted as the targets of lncRNA *MSTRG.42019*. In addition, many non-DE genes were targeted by lncRNA *MSTRG.42019*. Furthermore, the KEGG pathway search showed that the DEGs *TPM3*, *RSPO3*, *ACADVL*, and *CHRNA6* were mapped to specific pathways.

## 4. Discussion

In this study, we predicted 5864 novel lncRNA transcripts from the six skeletal muscle libraries, and our data showed that the characteristics of exon number, length distribution, and genomic expression level of these lncRNAs are similar to the previous report [35] and distinguished with the mRNAs. These predicted novel lncRNAs may play an important role in skeletal muscle growth and development, but the specific function still needs to be further explored. Moreover, 92 DE lncRNAs were identified between Bf and Sol from the RNA-seq analyses. Bf mainly composed of type IIB muscle fiber is a type of fast-twitch muscle, whereas Sol enriched in type I muscle fiber is a type of slow-twitch muscle [18]. Thus, these DE lncRNAs are the key candidates for the regulation of skeletal muscle fiber types. Certain types of skeletal muscle fiber are known to play a critical role in determining the meat quality postmortem in livestock, such as pH, meat color, and drip loss [6]. Thus, the DE lncRNAs identified in this study are the promising candidates influencing meat quality traits. Moreover, it is known that the alteration of muscle fiber types leads to the disorder of muscle physiological and metabolic properties, and thereby may cause many human diseases, such as muscular dystrophy, cardiomyopathic disease, and type 2 diabetes [2,3,4,5]. Thus, our data might provide some insights into human muscle diseases.

To understand the potential function of the identified lncRNAs, the target genes of DE lncRNAs were subjected to functional enrichment analyses. In the BP category, we found that the ATP metabolic process is one of the significantly enriched GO terms. ATP is the energy currency of living cells. Many biological processes are depended on the energy of ATP hydrolysis, for instance, the chromatin-structure dynamics critical for the regulation of gene expression and the chromosome function relies on ATP-dependent chromatin-remodeling complexes [36,37]. Skeletal muscle growth and development are also ATP-dependent, where the ATP induces muscle hypertrophy in slow muscle Sol via activating mTOR signaling pathway [38]. Furthermore, ATP is involved in the regulation of muscle fiber types and glucose metabolism by activation of P2Y receptors, phosphatidylinositol 3-kinase, Akt, and AS160 [39]. However, the regulatory mechanisms of lncRNAs on ATP metabolism and the effect of the lncRNA-ATP signaling pathway on skeletal muscle growth and muscle fiber conversion are still unknown.

Our KEGG pathway analysis showed that the cardiac muscle contraction pathway is significantly enriched. This result suggests that the DE lncRNAs identified in the skeletal muscle also play an important role in myocardial development. In addition, the glycolysis/gluconeogenesis, closely related to skeletal muscle fiber types, is significantly enriched. Moreover, fatty acid metabolism and fatty acid degradation pathways are also significantly enriched, while recent studies found that the change of fatty acid composition was accompanied by the transformation of skeletal muscle fiber types [40,41]. Thus, it will be interesting to investigate the specific regulatory mechanisms of the skeletal muscle fiber types and fatty acid composition mediated by the DE lncRNAs identified in this study. Different skeletal muscle fibers exhibit different glucose metabolic properties, while the pyruvate metabolism pathway critical for glucose metabolism is significantly enriched. The significantly enriched oxidative phosphorylation is the major source of ATP, while ATP is essential for many biological processes, including the growth and development and the fiber types of skeletal muscle [38,39]. Therefore, it is intriguing to investigate the potential mechanisms of the oxidative phosphorylation pathway regulating muscle fiber types which are mediated by the DE lncRNAs identified in this study. Furthermore, Alzheimer’s disease, Huntington’s disease, and Parkinson’s disease are three of the most significantly enriched pathways, thus the relationship between the skeletal muscle fiber types and these diseases is worth exploring.

To screen the key candidate lncRNAs affecting skeletal muscle fiber types, we performed the overlapped analysis of DE lncRNAs from Bf28 vs. Sol28, Bf35 vs. Sol35, Bf36 vs. Sol36, and Bf vs. Sol and identified 53 overlapped DE lncRNAs. Sol muscle (slow muscle) and Bf muscles (fast muscle) are two different skeletal muscles with various muscle fibers and have different physiological and metabolic properties, such as glycolytic potential. Therefore, these overlapped DE lncRNAs are most likely to regulate the formation of different muscle fiber types and participate in the regulation of glucose metabolism in skeletal muscle. Intriguingly, among these DE lncRNAs, we found that lncRNA *MSTRG.42019* is highly expressed in skeletal muscle and its expression is significantly higher in Sol muscle than in Bf muscle, suggesting that lncRNA *MSTRG.42019* may contribute to skeletal muscle fiber type conversion. Moreover, we found that the expression patterns of lncRNA *MSTRG.42019* were shown to up-regulate before birth from 110 days of pregnancy (P110) to the day of birth (D0) and then down-regulate after birth. However, the complete expression pattern of lncRNA *MSTRG.42019* at different developmental stages needs to be verified using more stages. Overall, our data suggest that lncRNA *MSTRG.42019* may participate in the regulation of skeletal muscle growth and development and are involved in skeletal muscle fibers conversion. However, understanding of the specific regulatory mechanisms warrants further study.

The above studies suggest that lncRNA *MSTRG.42019* is a promising lncRNAs affecting skeletal muscle fiber types, while skeletal muscle fibers are closely related to meat quality traits [42]. Therefore, we subsequently performed a correlation analysis between the lncRNA *MSTRG.42019* expression and meat quality traits and skeletal muscle fiber-related marker genes, *MYH7*, *MYH4*, and *MyoB*. Our results showed that lncRNA *MSTRG.42019* expression is significantly positively correlated with the *MYH7* expression, which is consistent with the expression of lncRNA *MSTRG.42019* higher in the Sol muscle (slow muscle) than that in the Bf muscle (fast muscle). These results suggest that lncRNA *MSTRG.42019* may regulate the formation of slow muscle fiber. Furthermore, we observed that the lncRNA *MSTRG.42019* expression is significantly correlated with glycolytic potential and drip loss. The slow muscle fibers (type I) display stronger oxidative metabolism capacity. On the other hand, fast muscle fibers (type IIb) exhibit higher glycolytic capacity, which is in accordance with the result of the lncRNA *MSTRG.42019* expression level significantly negatively correlated with glycolytic potential. The glycolytic potential is critical for pH decline postmortem, in turn, the change of pH is closely related to drip loss [43,44]. The change of pH in slow muscle with lower glycolytic potential postmortem is relatively moderate when compared with fast muscle with higher glycolytic potential, and the drip loss in slow muscle is relatively lower. The lncRNA *MSTRG.42019* expression level is higher in slow muscle than fast muscle, which is consistent with the result of its expression negatively correlated with the drip loss.

The predicted interaction network is very helpful for further study of the potential regulatory mechanism. In this study, we constructed a network map using the lncRNA *MSTRG.42019* and its target genes. *Parvalbumin* (*PVALB*) is the only down-regulated gene targeted by lncRNA *MSTRG.42019* in the interaction network. PVALB, a Ca^2+^ binding protein, is known to play a key role in muscle relaxation and it acts as an intracellular Ca^2+^ buffer to determine the duration and magnitude of the activating Ca^2+^ signal and the force and duration of contraction [45,46]. *PVALB* is positively associated with relaxation speed in skeletal muscle, but less PVALB expressed in the slow-twitch skeletal muscles, cardiac, or smooth muscle [47]. Moreover, ectopic expression of *PVALB* by injection of its cDNA into the slow-twitch muscle of rats leads to an increased relaxation rate [48]. Conversely, the fast-twitch muscle of the *PVALB*-knockout mouse showed a slower Ca^2+^ transient and relaxation rate than the wild-type mouse [49]. Since our result showed that lncRNA *MSTRG.42019* is highly expressed in Sol, we speculated that lncRNA *MSTRG.42019* could promote the formation of slow muscle fiber via Ca^2+^ signaling pathway mediated by *PVLAB*. Moreover, Glycerol-3-phosphate acyltransferase (GPAT) is a limited enzyme that can acylate the glycerol 3-phosphate in the first committed step in triacylglycerol (TAG) synthesis phosphate pathway [50]. Therefore, lncRNA *MSTRG.42019* may affect the intramuscular fat content and fatty acid composition by up-regulating *GPAT* expression. *Tropomyosin-3* (*TPM3*) is required for the early steps of myofibril formation in zebrafish [51] and our result showed that porcine lncRNA *MSTRG.42019* is highly expressed in P110, which suggests that the effects of lncRNA *MSTRG.42019* on embryonic or fetal skeletal muscle development might be associated with the *TPM3*. In addition, lncRNA *MSTRG.42019* also may up-regulate the expression of *RSPO3*, *HSPB6*, *LGI1*, *CHRNA6*, *SL35F1*, *ACADVL*, and *MAST2*. Furthermore, the KEGG pathway search showed that the DEGs *TPM3* involves in cardiomyopathy, *RSPO3* participates in Wnt signaling pathway, and *ACADVL* is critical for fatty acid metabolism. However, the relationship between lncRNA *MSTRG.42019* and its target genes, the exact mechanisms of lncRNA *MSTRG.42019*-related growth and development, and the conversion of the fiber types of skeletal muscle still need to be further explored.

In addition, many non-DE genes are targeted by lncRNA *MSTRG.42019*, such as *Cytochrome C Oxidase Subunit 5A* (*COX5A*). *COX5A* encodes a subunit of the respiratory chain complex IV (cytochrome c oxidase) [52] and its expression is associated with niacin-induced changes in fiber type distribution and expression of MHC isoforms. Moreover, we found that lncRNA *MSTRG.42019* targets several ATPase and ATP synthetase genes, including *ATP1A2*, *ATP2B2*, *ATP5E*, *ATP5I*, and *ATP5O*. These results were consistent with the results that the ATP metabolic process is significantly enriched in GO analysis, suggesting that lncRNA *MSTRG.42019* may play a pivotal role in energy metabolism. Collectively, our data provide preliminary evidence that lncRNA *MSTRG.42019* may play an important role in muscle fiber type specification, mitochondrial respiration activity, and energy metabolism.

## 5. Conclusions

We identified 92 DE lncRNAs between Bf and Sol which are promising candidates regulating skeletal muscle fibers and muscle fiber-related metabolic processes and found some potential signaling pathways related to skeletal muscle fiber formation and metabolic properties mediated by these DE lncRNAs, such as ATP metabolic process pathway, fatty acid metabolic pathway, and pyruvate metabolism. Furthermore, we identified that a skeletal muscle highly expressed DE lncRNA *MSTRG.42019* and documented that it is closely associated with meat quality traits. Overall, our data provide a solid foundation for an in-depth investigation of the lncRNA regulatory mechanisms of skeletal muscle growth and development and skeletal muscle fiber conversion.

## Supplementary Materials

The following are available online at https://www.mdpi.com/2073-4425/11/8/883/s1, Figure S1: Venn diagram of non-coding potential novel lncRNAs. The coding potential of novel lncRNAs was predicted using CNCI, CPC, PFAM, CPAT analysis. Figure S2: Characteristics of lncRNAs and mRNAs. (A) Length distribution of lncRNAs and mRNAs. (B) The number of lncRNAs and mRNAs exon. (C) Distribution of lncRNAs and mRNAs expression. Table S1: Primers used for qRT-PCR analysis. Figure S3: Expression patterns of lncRNA *MSTRG.42019* in Bf and Sol muscles, different tissues of adult pigs, and *Longissimus dorsi* muscles derived from different developmental stages, *n* = 3. Expression levels were determined by qRT-PCR and relative expression levels were calculated using the 2^−ΔΔct^ method and normalized to the expression of *HPRT*. The expression of lncRNA *MSTRG.42019* in the sample of the first column was determined as control and normalized to 1. All data are presented as mean ± SE. Figure S4: Correlation analyses between the expression of lncRNA *MSTRG.42019* and genes and meat quality traits. Correlation between the expression of lncRNA *MSTRG.42019* and *MyoB* (A), *MYH4* (B), carcass weight (C), back fat (D), pH_45min_ (E), *L*_24h_ (F), *a*_24h_ (G), *b*_24h_ (H), and intramuscular fat (I), respectively. Twenty *Longissimus dorsi* muscles were derived from a 279 [(P) × (D)] × [(L) × (Y)] commercial hybrid pig population. Table S2: Novel predicted lncRNA transcripts; Table S3: Genomic expression analysis of all lncRNA genes; Table S4: DE lncRNAs from Bf and Sol groups; Table S5: DE mRNAs from Bf and Sol groups; Table S6: Target gene prediction of lncRNAs; Table S7: GO and KEGG enrichment analyses; Table S8: 53 DE lncRNAs.

## Abbreviations

Bf: *Biceps femoris*; Sol: *Soleus*; DE: differentially expressed; GO: Gene Ontology; KEGG: Kyoto Encyclopedia of Genes and Genomes; lncRNAs: long non-coding RNAs. *MYH4*: *Myosin heavy chain 4*; *MYH7*: *Myosin heavy chain 7*.
