# Supplementary figures and images for: Exploring the lncRNAs Related to Skeletal Muscle Fiber Types and Meat Quality Traits in Pigs
Source: Genes (Basel). 2020 Aug 4;11(8):883. doi: 10.3390/genes11080883 (PMC7465969; doi:10.3390/genes11080883)

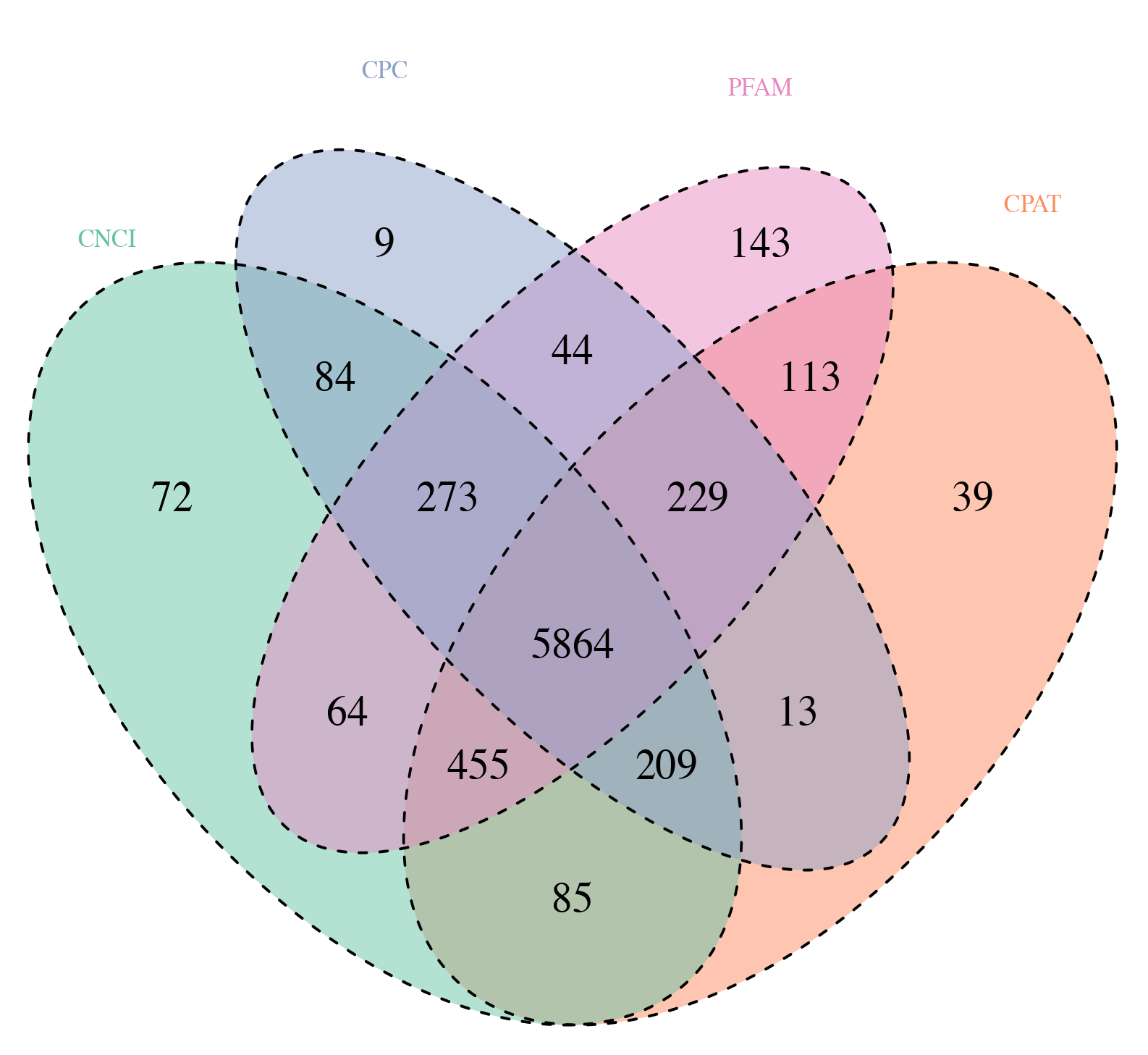

Supplement: Supplementary file 1 [file genes-11-00883-s001.zip › Additional file 1 Figure S1.tif]

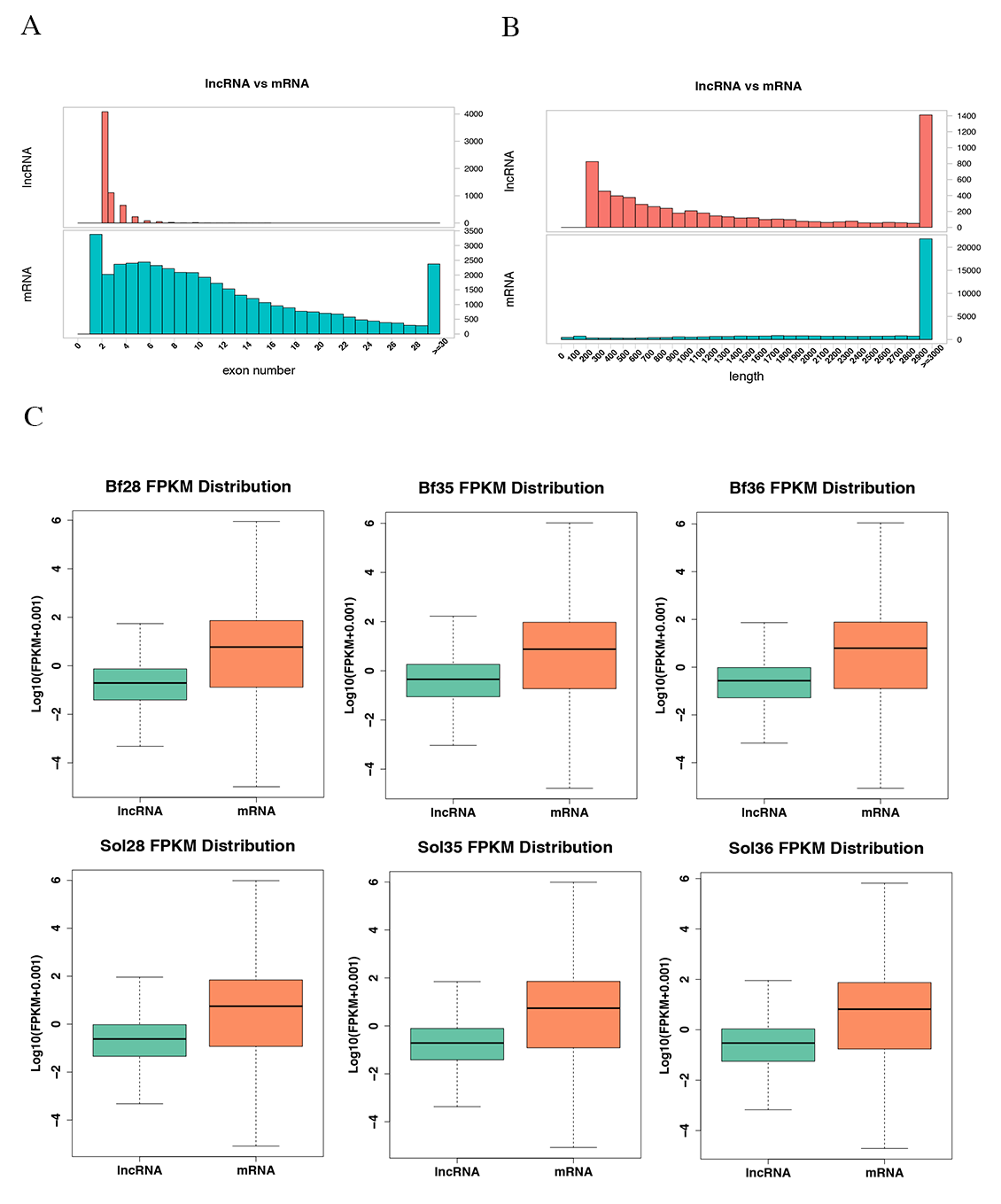

Supplement: Supplementary file 1 [file genes-11-00883-s001.zip › Additional file 2 Figure S2.tif]

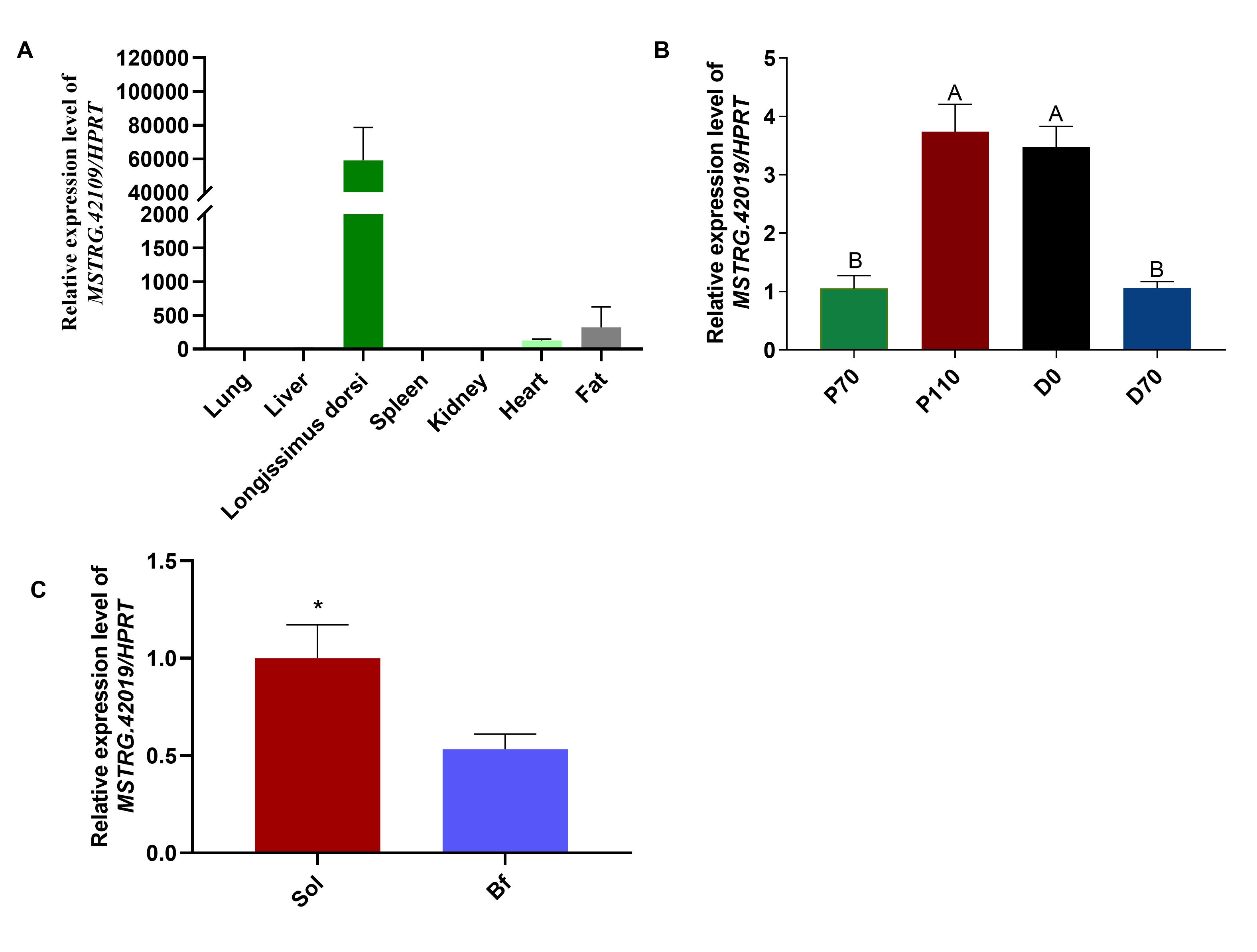

Supplement: Supplementary file 1 [file genes-11-00883-s001.zip › Additional file 3 Figure S3.tif]

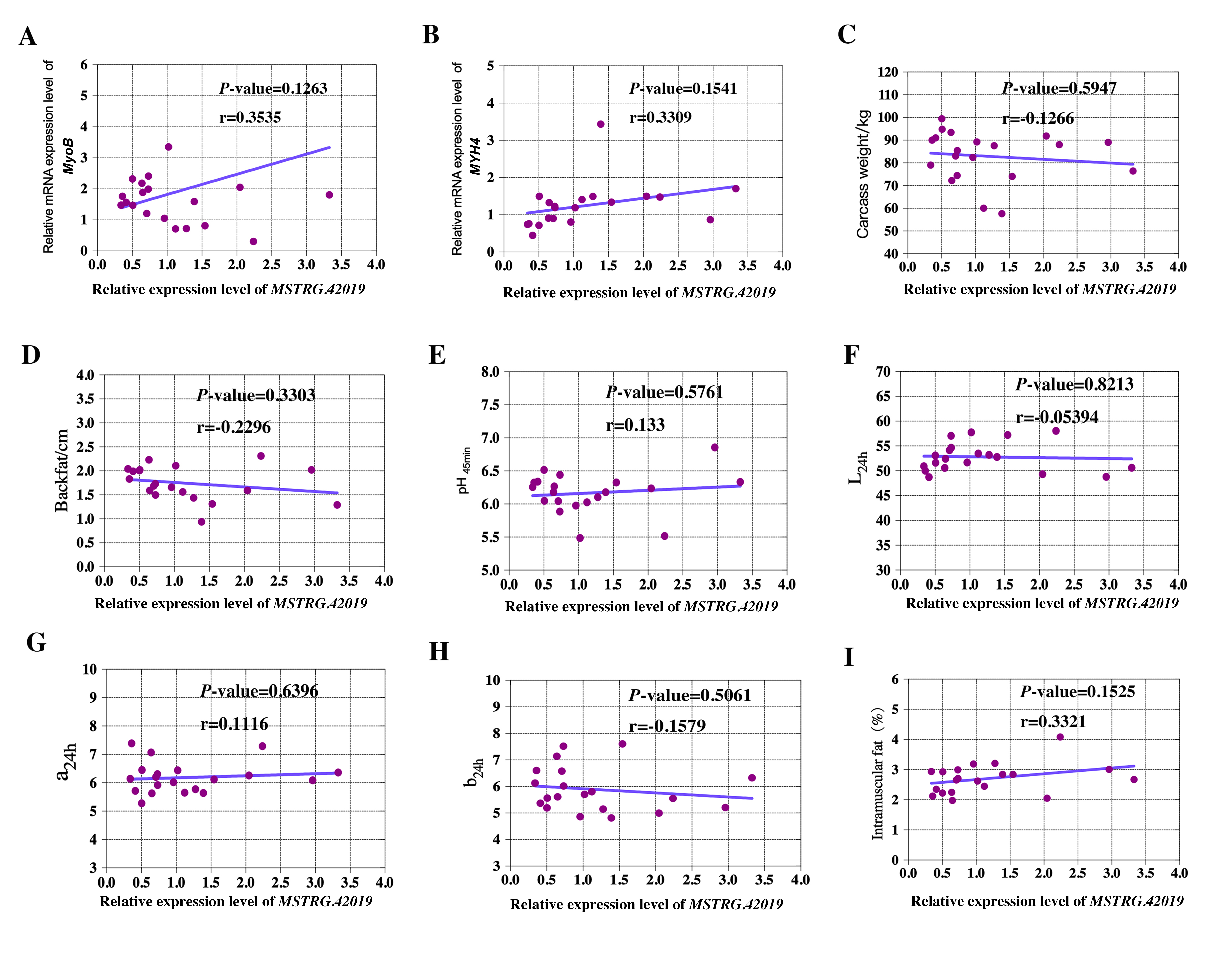

Supplement: Supplementary file 1 [file genes-11-00883-s001.zip › Additional file 4 Figure S4.tif]
