# Supplementary material for: Exploring the lncRNAs Related to Skeletal Muscle Fiber Types and Meat Quality Traits in Pigs
Source: Genes (Basel). 2020 Aug 4;11(8):883. doi: 10.3390/genes11080883 (PMC7465969; doi:10.3390/genes11080883)
Supplement: Supplementary file 1 [file genes-11-00883-s001.zip › Additional file 5 Table S1 Primers used for qRT-PCR analysis.doc]

| **Table S1. Primers used for qRT-PCR analysis** | | |
| --- | --- | --- |
| **lncRNAs** | **Forward primer** | **Reverse primer** |
| ENSSSCG00000033943 | GCTGTGTTGGAGAATGGA | CCTCTGCTGTGACTGTAG |
| ENSSSCG00000039815 | ATCCGTCAAGCACTATATCC | GTCGTTGTCACTCCATCC |
| MSTRG.92474 | GTTGTGACTGCTGTGGTT | CTGGACGGCTAAGAGGAT |
| MSTRG.80776 | CACCAGCAACGGATAAGA | CCACCATGAGATACTACTACA |
| MSTRG.135017 | GCCGTGAGTTGTTGTGTA | GATGAAGATGAAGGTAGAAGAC |
| MSTRG.18853 | GCTCTGCTGTTCCATTCA | CGCTGCTAATACTTCTATATCC |
| MSTRG.135005 | TAGCACTTCCTCTGTGTTAG | GAGGTAAGGTGAGGTAAGC |
| MSTRG.153756 | GCCACACTTGCTACACTT | ACGCTGATGAGGAGACAA |
| MSTRG.63150 | AAGGTCTGCTGAGGTTGT | CGTTGCTGTGGAATCTGT |
| MSTRG.42019 | GACGCAGGATAACAATAACA | CCAGCCAATCAAGAGAAGA |
| MSTRG.35730 | CAAGCACGACTACAGCACCA | TGACTGCCATAATCCAGGCT |
| MSTRG.43472 | CAAAGCCGATGACTTGACGC | GCAGAAGTGAAGTTCGCAAGG |
| Myogloblin | GGATGAGATGAAGGCCTCTG | CCTCTCATGAACAAAGCAAC |
| MyHCI | CGACACACCTGTTGAGAAG | AGATGCGGATGCCCTCCA |
| MyHCIIB | GTTCTGAAGAGGGTGGTAC | AGATGCGGATGCCCTCCA |
| GAPDH | GGGCATGAACCATGAGAAGT | AAGCAGGGATGATGTTCTGG |
